# Adherence to National Antimicrobial Guidelines in Hospitalized Geriatric Patients with Community-Acquired Pneumonia: A Prospective Observational study in a Malaysian Hospital

**DOI:** 10.3390/antibiotics10121490

**Published:** 2021-12-04

**Authors:** Sadia Shakeel, Jaya Muneswarao, Azrina Abdul Aziz, Heng Yeong Le, Fatin Syazwanni Abd. Halim, Anees Ur Rehman, Rabia Hussain

**Affiliations:** 1 Department of Pharmacy Practice, Faculty of Pharmaceutical Sciences, Dow College of Pharmacy, Dow University of Health Sciences, Karachi 74200, Pakistan; sadia.shakeel@duhs.edu.pk; 2 Discipline of Social and Administrative Pharmacy, School of Pharmaceutical Sciences, Universiti Sains Malaysia, Penang 11800, Malaysia; aneesurrehmanr90@gmail.com; 3 Pharmacy Department, Hospital Pulau Pinang, Ministry of Health Malaysia, Penang 10990, Malaysia; rahulraoraorao@gmail.com; 4 Pharmacy Department, Hospital Kulim, Ministry of Health Malaysia, Kedah 09090, Malaysia; azrina.aa@gmail.com (A.A.A.); nssl036@gmail.com (H.Y.L.); fatinsyazwanni90@yahoo.com (F.S.A.H.); 5 Department of Pharmacy Practice, Faculty of Pharmacy, Bahauddin Zakariya University, Multan 60800, Pakistan

**Keywords:** adherence, CURB-65, community-acquired pneumonia, empiric antibiotics, standard treatment guidelines, Malaysia

## Abstract

The evaluation of disease progression and onsite therapeutic care choices for community-acquired pneumonia (CAP) patients is vital for their well-being and the optimum utilization of healthcare resources. The current study was conducted to assess physicians’ adherence to clinical practice standards and antibiotic prescribing behavior for the treatment of CAP in older people. A prospective study that included 121 consecutive patients admitted for CAP was conducted at Kulim Hospital, Kedah, from March 2020 to August 2020. Medical records including demographic data, comorbidity, physical examination, laboratory or radiologic findings, and drugs used for the treatment of CAP were accessed from bed head tickets (BHT). The mean age for patients was 73.5 ± 6.2 years, 73 (60.3%) and 48 (39.6%) were males and females, respectively. Amoxicillin/clavulanate (19.8%) was the most prescribed antibiotic for non-severe pneumonia followed by ampicillin sodium/sulbactam sodium (6.6%), while in patients with severe CAP beta-lactam + beta lactamase inhibitors (BLIs) with a combination of macrolide were the most common antibiotics prescribed either in patients with (21.4%) or without co-morbidities (8.2%). The average length of stay in the hospital with severe pneumonia was 6–7 days for 23.9% of patients and < 5 days for 21.4% of patients. The duration of intravenous antibiotics in patients with severe pneumonia was 6–7 days for 32.2% of patients. The present findings revealed the adherence of antibiotic prescribing practices to the Malaysian National Antimicrobial Guideline 2019 for CAP therapy among geriatric patients and adherence to the CAP criteria for hospital admissions.

## 1. Introduction

Community-acquired pneumonia (CAP) is one of the most prevalent infections globally, with a mortality rate of greater than 50% in hospitalized patients and 1% in outpatient settings [1]. Inapt management of patients or postponement of patients’ admittance to an intensive care unit (ICU) were revealed to accompany augmented mortalities [2]. It is challenging to make a clinical diagnosis of pneumonia in the elderly, as the typical signs of pneumonia are less common in older patients [3]. Aging causes changes in basic lung physiology such as decreased elastic recoil, increased air trapping (senile emphysema), decreased chest wall compliance, and decreased respiratory muscle strength [4]. These factors may raise the baseline effort of breathing, leaving older people with less reserves with which to deal with bacterial infections in the lungs. Furthermore, decreased mucocilliary clearance and cough reflex have been reported [5,6]. These findings, in conjunction with higher upper airway colonization with pathogenic microbes, may predispose this population to lower respiratory tract infections. Furthermore, multiple age-related variables, such as comorbidities, nutritious conditions, and swallowing difficulties, have all been linked to an increase in the prevalence of CAP in the elderly [2]. *Streptococcus pneumonia* is the most prevalent infection among the old, but aspiration pneumonia and drug-resistant bacteria can also cause CAP [3]. Despite the adoption of the 23-valent pneumococcal polysaccharide vaccine, the burden of pneumococcal CAP in adults remained high. It leads to increased rates of hospitalization and duration of stay, particularly among elderly individuals with comorbidities [4]. CAP is responsible for 98.8 episodes per 10,000 discharges in Malaysia [5].

The mortality between these vulnerable groups greatly depends on the clinical settings where they are treated [3]. The early detection of patients suffering from severe pneumonia is significant for general practitioners [5]. An accurate diagnosis, as well as judicious antibiotic administration and usage, are critical for reducing the issue of antibiotic resistance. When first-line antibiotics are no longer effective in treating infections, owing to microbial resistance, more costly medications need to be employed. Longer sickness and treatment, frequently in hospitals, raise healthcare expenses as well as the economic strain on families and society [6]. The most effective recommendations for the care of CAP patients are vaccination and case management, according to the World Health Organization (WHO) [7]. These have been demonstrated to reduce mortality and morbidity, thereby reducing hospital stays [7,8]. Several studies have found that following treatment guidelines and adhering to them reduces morbidity, death, and healthcare expenditures [8,9]. Although guidelines can steer and standardize illness care, their influence on disease outcomes is less quantifiable. Adherence to current CAP recommendations improves treatment outcomes in older patients significantly [10]. In this category of fragile patients, special care should be devoted to nutritional conditions, fluid administration, clinical outcomes, and comorbidity therapy. However, data suggest that national recommendations for the care of CAP patients are commonly ignored in clinical practice [8]. 

The ATS/IDSA (American Thoracic Society and Infectious Diseases Society of America) guidelines for CAP treatment were developed to assist physicians in CAP management and standardize medical care [11]. These guidelines seek to reduce morbidity and death rates in CAP patients by improving their clinical treatment. National antimicrobial guidelines (NAGs) were developed to support reasonable antibiotic usage by physicians, pharmacists, and all healthcare professionals, which can help to reduce antimicrobial resistance (AMR) and healthcare costs [12]. According to implementation science, variables influencing healthcare experts’ adherence to management guidelines can be associated with the context in which treatment and care are provided [13]. While numerous pieces of research highlight impediments to guideline adoption, there is no practical guidance on how to translate these findings into clinical-practice-changing initiatives. Some researchers have identified potential hurdles and problems to healthcare workers adhering to treatment guidelines [13]. Physicians have an important role in the care of CAP by providing a prompt diagnosis, a suitable antibiotic regimen, and identifying risk factors. Discrepancies between prescribed treatments and actual management techniques by physicians are frequently noticed, highlighting the necessity to evaluate our local clinical practices [14]. There has been insufficient research on the adherence to treatment guidelines for CAP; the current study is the first to assess physicians’ adherence to clinical practice standards and antibiotic prescribing behaviors for the treatment of CAP in older people in Hospital Kulim, Kedah.

## 2. Results

### 2.1. Patients’ Baseline Characteristics

Consecutively, 121 patients with a diagnosis of CAP were prospectively analyzed. Table 1 depicts the patients’ baseline characteristics. The mean age for the study-enrolled patients was 73.5 ± 6.2 years; *n* = 73 (60%) of the patients were males and *n* = 48 (40%) were females. The majority, *n* = 89 (73%), of the patients were Malay, followed by Chinese, *n* = 17 (14%), and Indians, *n* = 15 (12%). Slightly more than half, *n* = 65 (54%), were living in rural areas and *n* = 65 (54%) were retired from their jobs. Only *n* = 46 (38%) were smokers. The most commonly observed co-morbidities were hypertension, *n* = 85 (70%), DM, *n* = 59 (49%), and COPD, *n* = 32 (26%). The majority of the patients (*n* = 113; 93%) were evaluated using the CURB-65 score recommended in national guidelines for determining the severity of pneumonia and subsequent treatment.

### 2.2. CAP Criteria for Hospital Admission and Patients’ Outcomes

The patients were examined for baseline O_2_ saturation, RR, HR, platelet count, SBP or DBP, albumin, serum lactic acid dehydrogenase (LDH), and urea. According to the severity level, *n* = 57 (47.1%) had mild-to-moderate CAP. In comparison, *n* = 64 (52.8%) patients were hospitalized for severe CAP based on the physician’s diagnosis. 

Some laboratory findings were done within 24 h of hospital admission: RR ≥ 30/min was present in 88 patients, HR ≥ 125/min was observed in 12 patients, platelet count < 100 × 10^3^ /mm^3^ was present in 15 patients, SBP < 90 mmHg or DBP ≤ 60 mmHg was found in 27 patients, albumin < 3.5 g/dl was observed in 57 patients, serum LDH > 230 U/L was found in 75 patients, and urea > 7 mmol/L was present in 50 patients (Table 2). Regarding patients’ outcomes: the mean length of stay (LOS) was 4.5 ± 3.3 days and the rate of ICU admittance as well as overall 30-day mortality was 37.1% and 10.7%, respectively. 

### 2.3. Empiric Antibiotic Prescribed for CAP Patients

According to our findings, the antimicrobial regimens prescribed for the geriatric patients were consistent with the recommendations in the Malaysian National Antibiotic Guideline [12]. Amoxicillin/clavulanate plus azithromycin are the preferred antibiotics whereas ceftriaxone plus azithromycin is an alternative suggested treatment for CAP in adults. Levofloxacin is strictly reserved for patients who were allergic to penicillin due to higher chances of adverse reactions [12]. Table 3 depicted the different antibiotic regimens that were prescribed for CAP patients. Beta lactam + BLIs (*n* = 32; 26.4%), including amoxicillin/clavulanate, 1.2 gm IV q8h (*n* = 24; 19.8%), followed by ampicillin sodium/sulbactam sodium, 1.5 gm IV q8h (*n* = 8; 6.6%), were the most commonly prescribed antibiotics for non-severe pneumonia, while in patients with severe CAP, beta lactam + BLIs with a combination of macrolide (azithromycin; 500 mg IV/PO q24h) were the most common antibiotics prescribed either in patients with (*n* = 26; 21.4%) or without co-morbidities (*n* = 10; 8.2%). Ceftriaxone, 2 gm IV q24h, was the antibiotic used in patients with co-morbidities either alone or in combination with azithromycin.

### 2.4. Patients’ Duration of CAP Hospitalization and Intravenous Antibiotic Use

Table 4 depicts patients’ duration of CAP hospitalization and intravenous (IV) antibiotic use. The average length of stay in the hospital with non-severe pneumonia was 3–5 days for *n* = 22 (18.1%) patients and < 3 days for *n* = 14 (11.5%) patients. The average length of stay in the hospital with severe pneumonia was 6–7 days for *n* = 29 (23.9%) patients and < 5 days for *n* = 26 (21.4%) patients. The duration of IV antibiotics to a patient with severe pneumonia was 6–7 days for *n* = 39 (32.2%) patients.

### 2.5. Management of Therapeutic Failure

Patients who did not show clinical improvement within 72 h were classified as non-responders. Instability of the hemodynamic system, respiratory function impairment, and radiographic progression were considered to be markers of therapeutic failure, and mechanical ventilation was suggested for these patients. 

### 2.6. Criteria for Discharge

**Figure 1:**
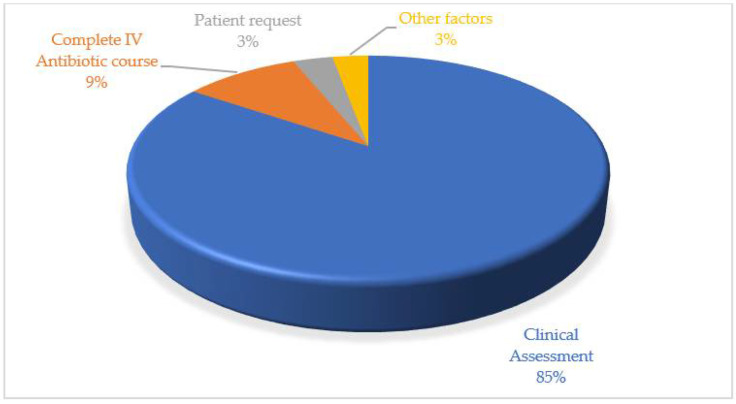
Physicians’ criteria for discharging CAP patients.

Clinical assessment (85%) followed by a complete course of IV antibiotics (9%) were the major factors observed to discharge a patient from the hospital (Figure 1). The major clinical assessment factors were a reduced fever for at least 12–24 h, a normal activity level, a healthy appetite, stable mental status, and consistent pulse oximetry measurements of > 90% in ambient air for at least 12–24 h.

## 3. Discussion

To the best of our knowledge, this is the first study in Malaysia that looks into physicians’ adherence to the standard management guiding principles for the diagnosis and management of CAP geriatric patients. CAP is a frequent diagnosis that incurs considerable healthcare expenditures, particularly for individuals who require hospitalization, and it is one of the most common diseases for which antibiotics are administered [15]. The diagnostic and therapeutic complexity of moderate-to-severe frail elderly patients is high, and it encompasses variables that may influence the etiology, diagnostic and invasive treatment procedures, and the patient’s final placement. These patients typically have significant comorbidities and polypharmacy, rendering them more susceptible to the emergence of adverse drug responses [16]. According to the findings of the study, there was an adherence to the treatment standards of CAP and the practice of using severity scores in the assessment to admit patients to the hospital. As the Malaysia National Antimicrobial Guideline 2019 management recommendations are available to clinicians, CAP diagnoses based on the severity score utilizing the CURB-65 were found in this investigation [12]. The majority of the patients (93%) were evaluated using the CURB-65 score, which is recommended in national guidelines for determining the severity of pneumonia and subsequent treatment. In previous studies, low usage of a pneumonia severity score was observed [17,18]. Furthermore, numerous investigations have shown that the CAP severity score was recorded incorrectly [19]. The CURB-65 score assists clinicians in classifying patients and selecting appropriate antibiotic treatment based on the patient’s condition. The severity scores of CAP have been shown to enhance care for CAP patients by serving as independent predictors of illness severity. Execution strategies increase the adherence rates, which are needed for patient safety and health expenditures [20].

According to the severity level, 57 individuals (47.1%) had mild-to-moderate CAP. In comparison, 64 (52.8%) patients were hospitalized for severe CAP based on the physician’s diagnosis. According to one study, when the CURB-65 score was not used, between 50 and 78 % of individuals with CAP were hospitalized needlessly [21]. The cost for CAP inpatient care is almost double that of outpatient care. Non-compliance by physicians to the standard care may have a significant financial influence on the healthcare system. Adherence to empiric antibiotic recommendations for the therapy of CAP patients has been shown to minimize mortality and morbidity, reduce hospital stays, and lower healthcare expenditures. Suitable antibiotic delivery in hospitals assures successful patient care, and the provision of suitable antibiotics within 4–8 h is associated with a 5–43% comparative decrease in mortality [22,23]. 

In patients hospitalized for CAP, the empiric antibiotic regimes are intended to treat *S aureus* and Gram-negative enteric bacilli (e.g., *Klebsiella pneumoniae*) in addition to typical pathogens (e.g., *S pneumoniae*, *H influenzae*, and *M catarrhalis*) and atypical pathogens (e.g., *Legionella pneumophilia*, *M pneumoniae*, and *C pneumoniae*). Therapy is started as soon as CAP is suspected as the diagnosis, ideally within 4 h of presentation [24]. In the present study, the majority of the empiric antibiotic prescriptions followed the national treatment guidelines. There is evidence that empiric antibiotic recommendations for the care of CAP patients frequently conform to the clinical practice. Over the last two decades, amoxicillin/clavulanate has been used to treat a variety of infections, including CAP. Several studies included in a review showed that amoxicillin/clavulanate had a high clinical success rate in respiratory infections [24]. Amoxicillin/clavulanate is commonly given for RTIs since the causal agents of these infections are not always identified, necessitating empirical treatment [25]. In the current study, amoxicillin/clavulanate was the most commonly recommended antibiotics by physicians for the treatment of CAP in hospitalized patients. The addition of azithromycin in non-severe and severe CAP is appropriate according to national antibiotic guideline recommendations [12]. While the use of cephalosporins such as ceftriaxone was found to be low in CAP treatment in this study, in one study ceftriaxone was administered to more than 72.4% of patients with CAP, either alone or in combination with azithromycin [17]. According to another piece of research, more than 92% of CAP patients were administered ceftriaxone in the hospital. Another study found that the usage of broad-spectrum cephalosporins in mild-to-moderate CAP patients was related to a high number of conflicting prescription events [26]. Similarly, several studies have raised concerns that the usage of third-generation cephalosporins is excessive and inconsistent with standard treatment guidelines [27]. Antibiotic misuse is associated with avoidable adverse medication effects and increased healthcare costs, and it contributes to the growing worldwide issue of antimicrobial resistance [10]. Furthermore, while certain new medicines are found to be effective in the treatment of CAP (delafloxacin, lefamulin), none of these drugs may be utilized in the vulnerable population [28]. Healthcare practitioners could play a role in educating the population in order to reduce the inappropriate use of antibiotics. In this study, the preferred method of antibiotics was IV, in contradiction to the findings of another study in which physicians chose oral antibiotics over IV or IM antibiotics [29]. It is documented that physicians believed broad-spectrum or new antibiotics to be more effective, which is an impression that is frequently reinforced by pharmaceutical organizations’ advertising. In the current study, the average length of stay in hospital in severe pneumonia was 6–7 days for 23.9% patients and < 5 days for 21.4% patients. The Malaysia National Antimicrobial Guideline recommendations indicate that CAP patients need to be treated for at least five to seven days [12]. It is observed that the majority of patients achieve clinical stability within three to four days of beginning antibiotic therapy. As a result, for patients who show a satisfactory clinical response during the first two to three days of therapy, the suggested length is generally five to seven days overall [30]. The normal schedule of seven to ten days may be appropriate unless there is a suspicion of *Pseudomonas* infection, in which case the therapy should be extended to 14 days. Other clinical circumstances that may necessitate extended antibiotic therapy include the persistence of a fever for more than 72 h, the persistence of more than one clinical instability criterion, insufficient initial coverage, or the development of sequelae [5]. Biomarkers such as procalcitonin and C-reactive protein may be beneficial in reducing the duration of antibiotic therapy [31]. Due to the significant mortality associated with CAP and the improbability of sufficient GI absorption of oral antibiotics in very sick patients, it is generally preferred to administer IV antibiotics at the outset of therapy for patients hospitalized for CAP. When a patient’s clinical condition improves, IV antibiotics can be moved to oral treatment [32]. IV antibiotics administration to a patient with severe pneumonia was 6–7 days for 32.2% patients. With adequate antibiotic medication, a patient’s clinical condition typically improves within 48 to 72 h [33]. In the present study, the patients who did not show clinical improvement within 72 h were classified as non-responders. Clinical assessment (85%) followed by a complete course of IV antibiotics (9%) were the major factors observed to discharge a patient from the hospital. An early discharge, depending on clinical stability and criteria for switching to oral treatment, is advocated to avoid needless hospital expenditures and hazards, such as iatrogenic consequences and an increased risk of antibiotic resistance [34]. 

There are some limitations in the current study. First, a comparatively small number of patients were involved in the study. Secondly, this study did not investigate the etiological diagnosis of the patients involved to recognize underlying organisms and their relationship with risk elements of mortality. The other limitations include the fact that the assessments were based on an observation at a single point in time completed over six months and that the study was not a multi-sectoral observational study.

## 4. Materials and Methods

### 4.1. Study Population and Settings

An observational prospective research on consecutive adult patients (aged 65 years or older) admitted from March 2020 to August 2020 at Hospital Kulim Kedah was carried out. The hospital is a public sector hospital situated in Kulim, Kedah Darulaman, Malaysia, and is funded by the federal government. The patients were involved in the study for the duration of their length of stay in the hospital. The ward pharmacists identified these patients based on the patients’ registry daily. 

The authors had no say in the hospitalization decision, which was solely made by consulting physicians. Patients with hospital-acquired pneumonia, aspiration pneumonia, chronic pulmonary illness exacerbations, pulmonary tuberculosis, and those who were immunocompromised were not included in the research. Patients who were not given antibiotics on the first day of their stay were likewise eliminated. 

### 4.2. Instruments and Techniques for Data Collection

The CURB-65 score (C—confusion; U—uremia or blood urea nitrogen (BUN); R—respiratory rate; B—blood pressure; and age more than 65) was used to categorize the severity and determine admission [14]. Each variable is given one point for a total possible score of five. Patients with a CURB-65 score of 1 or less were considered as outpatients. In the case of a score greater than one, the patient was admitted to the hospital. A patient with a high CURB-65 score was at a high risk of death. 

For eligible patients, a standardized questionnaire was utilized to collect the necessary information. The baseline patient assessment began on the first day of their hospitalization. The second and third assessments were completed within 48 h after admission and on the day of discharge, respectively. At assessment one, the socio-demographic information of the enrolled patients was gathered, which included age, gender, marital status, and occupational status. On admission and re-assessment, the following data were recorded: clinical signs and symptoms (body temperatures, respiratory rate, arterial blood pressure, and heart rate), partial oxygen saturation, radiographic screening, and antibiotic regimes. Comorbidities were noted in case the patient had one or more diseases, i.e., cerebrovascular diseases, chronic kidney disease, congestive heart failure, chronic liver disease, malignancy, diabetes mellitus, or chronic obstructive pulmonary disease (COPD). The laboratory test findings were those which were within 24 hours of patient admission. The patients were interviewed only if further information was required, for instance, the social history of smoking or alcohol intake. The subjects had the choice to withdraw at any time. The presence of specific clinical signs and symptoms, as well as investigations, are required for the diagnosis of CAP. 

The antibiotic administered within the first 24 h of admission was regarded as the initial therapy. The previous antibiotic regimen obtained in the outpatient setting for the present illness was likewise documented. When the antibiotics were prescribed by the physician followed the guidelines provided in the Malaysia National Antimicrobial Guideline [12], the antimicrobial regimen was considered as adhering to the guideline. If clinicians used CURB-65 criteria to decide on patients’ hospitalization and discharge, they were considered as adhering to the guideline.

### 4.3. Ethical Consideration

The study was registered in the National Medical Research Register (NMRR) database (NMRR-20-119-52658). Ethical approval was acquired from the Medical Research & Ethics Committee (MREC), Ministry of Health, Malaysia. Written informed consent from each patient was obtained before data collection. For those patients with severe cognitive impairment due to dementia, their health care proxy was invited to provide written informed consent. The consent form was provided after clarifying the rationale of the study. The informed consent form was written in both English and the simple local language easily understood by the patients to reduce the prospect of coercion or undue impact, and the patients were given adequate time to consider their contribution. A researcher was available at the site to answer queries of potential subjects.

### 4.4. Statistical Analysis

The collected responses were further investigated by the Statistical Package for Social Sciences (SPSS 24.0, Chicago, IL, US). The demographic profiles of the patients were recorded in percentages and frequencies. Descriptive analysis was used to calculate the proportions. Tables were used to present the data for easy interpretation and comprehension.

## 5. Conclusions

The present findings revealed the adoption of national guidelines in Malaysian hospitals. The adherence to CAP criteria for hospital admission and antibiotic selection was appropriate; however, the length of therapy and hospitalization may still be improved. The pneumonia severity rating tool was utilized to guide therapy or antibiotic prescriptions. The majority of the patients were hospitalized with appropriate reason and were rationally prescribed broad-spectrum antibiotics. The findings may be beneficial for local health authorities in establishing future efforts to homogenize and offer optimum patient care.

## Figures and Tables

**Table 1:** Patients’ baselines characteristics.

Baseline Characteristics		*n* (%)
Age (years)		
Mean ± SD	73.5 ± 6.2	
Median	72	
Range	64–90	
Height (cm, mean ± SD)	164 ± 8.8	
Weight (kg, mean ± SD)	59.7 ± 12.4	
Mean length of stay (days)	4.5 ± 3.3	
Gender	Male	73 (60)
	Female	48 (40)
Race	Malay	89 (73)
	Chinese	17 (14)
	Indian	15 (12)
Residence	Urban	56 (46)
	Rural	65 (54)
Occupation	Employed	2 (2)
	Unemployed	51 (42)
	Retired	65 (54)
	Own business	3 (2)
Smoker	Yes	46 (38)
	No	75 (62)

**Table 2:** Physical and laboratory findings at hospital admission and patients’ outcomes.

Age > 65 years	111 (91.7)
**BMI**	
Underweight	11 (9)
Normal	96 (79)
Overweight	12 (10)
Obese	2 (2)
SBP < 90 mmHg or DBP ≤ 60 mmHg	27 (22.3)
LDH > 230 U/L	75 (61.9)
Urea > 7 mmol/L	50 (41.3)
Platelet count < 100 × 10^3^ /mm^3^	15 (12.3)
Albumin < 3.5 g/dl	57 (47.1)
**Patients’ outcomes**	
LOS: mean ± SD (days)	4.5 ± 3.3
Cure	62 (51.2)
30-day mortality	13 (10.7)
ICU admission	45 (37.1)
Readmitted within 30 days	1 (0.8)

**Table 3:** Empiric antibiotics prescribed for CAP patients.

Scenario	Antibiotic Prescribed		
Non-Severe Pneumonia			
With no co-morbidities	Beta lactam + beta lactamase inhibitors (BLIs) *	Beta lactam + BLIs ***plus*** macrolide **	Doxycycline
	11 (9%)	3 (2.4%)	1 (0.8%)
With co-morbidities	Beta lactam+ BLI s ***plus*** macrolide	Ceftriaxone ***plus*** macrolide	Ceftriaxone
	21 (17.3%)	13 (10.7%)	8 (6.6%)
**Severe Pneumonia**			
With no co-morbidities	Beta lactam + BLIs	Beta lactam + BLIs ***plus*** macrolide	Beta lactam + BLIs ***plus*** doxycycline
	4 (3.3%)	10 (8.2%)	7 (5.7%)
With co-morbidities	Beta lactam+ BLIs ***plus*** macrolide	Ceftriaxone ***plus*** macrolide	Ciprofloxacin
	26 (21.4%)	12 (9.9%)	5 (4.1%)

* Amoxicillin/clavulanate/ampicillin sodium/sulbactam sodium; ** azithromycin.

**Table 4:** Patients’ duration of CAP hospitalization and intravenous antibiotic use.

Length of Hospitalization and IV Use of Antibiotics	No. of Days	Frequency (*n*; %)
Duration of hospital stay with non-severe pneumonia	< 3 days	14 (11.5)
	3–5 days	22 (18.1)
	6–7 days	5 (4.1)
	8–10 days	2 (1.6)
Duration of hospital stay with severe pneumonia	< 5 days	26 (21.4)
	6–7 days	29 (23.9)
	8–10 days	15 (12.3)
	11–14 days	8 (6.6)
Duration of IV antibiotics use in patients with severe pneumonia	< 3 days	13 (10.7)
	3–5 days	20 (16.5)
	6–7 days	39 (32.2)
	8–10 days	6 (4.9)
